# Factors associated with the onset of major depressive disorder in adults with type 2 diabetes living in 12 different countries: results from the INTERPRET-DD prospective study

**DOI:** 10.1017/S2045796020000438

**Published:** 2020-06-02

**Authors:** C. E. Lloyd, N. Sartorius, H. U. Ahmed, A. Alvarez, S. Bahendeka, A. E. Bobrov, L. Burti, S. K. Chaturvedi, W. Gaebel, G. de Girolamo, T. M. Gondek, M. Guinzbourg, M. G. Heinze, A. Khan, A. Kiejna, A. Kokoszka, T. Kamala, N. M. Lalic, D. Lecic-Tosevski, E. Mannucci, B. Mankovsky, K. Müssig, V. Mutiso, D. Ndetei, A. Nouwen, G. Rabbani, S. S. Srikanta, E. G. Starostina, M. Shevchuk, R. Taj, U. Valentini, K. van Dam, O. Vukovic, W. Wölwer

**Affiliations:** 1 The Open University, Milton Keynes, UK; 2 Association for the Improvement of Mental Health Programmes, Geneva, Switzerland; 3 Child Adolescent & Family Psychiatry, National Institute of Mental Health (NIMH), Dhaka, Bangladesh; 4 Servicio de Endocrinologia y Medicina Nuclear del Hospital Italiano de Buenis Aires, Buenis Aires, Argentina; 5 The Mother Kevin Post Graduate Medical School, Uganda Martyrs University, Kampala, Uganda; 6 Federal Medical Research Centre for Psychiatry and Narcology, Moscow, Russia; 7 Department of Neurosciences, Biomedicine and Movement Sciences, University of Verona, Verona, Italy; 8 National Institute of Mental Health & Neurosciences, Bangalore, India; 9 Department of Psychiatry and Psychotherapy, Medical Faculty, Heinrich Heine University of Düsseldorf, Düsseldorf, Germany; 10 Istituto Centro San Giovanni di Dio Fatebenefratelli, Brescia, Italy; 11 European Psychiatric Association – Early Career Psychiatrists Committee, Wroclaw, Poland; 12 Servicio de Psiquiatría del Hospital Italiano de Buenos Aires, Buenis Aires, Argentina; 13 Universidad Nacional Autónoma de México, Mexico City, Mexico; 14 Pakistan Institute of Medical Sciences, Islamabad, Pakistan; 15 WSB University, Toruń, Poland; 16 University of Lower Silesia, Wroclaw, Poland; 17 II Department of Psychiatry, Medical University of Warsaw, Warszawa, Poland; 18 Diabetes Centre and Jnana Sanjeevini Medical Centre, Bangalore, India; 19 Clinic for Endocrinology, Belgrade University School of Medicine, Serbian Academy of Sciences and Arts, Belgrade, Serbia; 20 Serbian Academy of Sciences and Arts, Belgrade, Serbia; 21 Diabetology – Careggi Teaching Hospital and University of Florence, 50141, Florence, Italy; 22 Department of Diabetology, National Medical Academy for Postgraduate Education, Kyiv, Ukraine; 23 Institute for Clinical Diabetology, German Diabetes Center, Leibniz Center for Diabetes Research at Heinrich Heine University of Düsseldorf, Düsseldorf, Germany; 24 Department of Endocrinology and Diabetology, Medical Faculty, Heinrich Heine University, Düsseldorf, Germany; 25 German Center for Diabetes Research (DZD), München-Neuherberg, Germany; 26 Africa Mental Health Research and Training Foundation, Nairobi, Kenya; 27 University of Nairobi, Africa Mental Health Research and Training Foundation, Nairobi, Kenya; 28 Middlesex University, London, UK; 29 Neurodevelopmental Protection Trustee Board, Dhaka, Bangladesh; 30 Samatvam Endocrinology Diabetes Centre and Jnana Sanjeevini Medical Centre, Bangalore, India; 31 Department of Endocrinology, Moscow Regional Clinical and Research Institute, Moscow, Russia; 32 Diabetology Unit, ASST Spedali Civili, Brescia, Italy; 33 School of Medicine, University of Belgrade, Institute of Mental Health, Belgrade, Serbia

**Keywords:** Depression, mental health, prospective study, psychological assessment, stress

## Abstract

**Aims:**

To examine the factors that are associated with changes in depression in people with type 2 diabetes living in 12 different countries.

**Methods:**

People with type 2 diabetes treated in out-patient settings aged 18–65 years underwent a psychiatric assessment to diagnose major depressive disorder (MDD) at baseline and follow-up. At both time points, participants completed the Patient Health Questionnaire (PHQ-9), the WHO five-item Well-being scale (WHO-5) and the Problem Areas in Diabetes (PAID) scale which measures diabetes-related distress. A composite stress score (CSS) (the occurrence of stressful life events and their reported degree of ‘upset’) between baseline and follow-up was calculated. Demographic data and medical record information were collected. Separate regression analyses were conducted with MDD and PHQ-9 scores as the dependent variables.

**Results:**

In total, there were 7.4% (120) incident cases of MDD with 81.5% (1317) continuing to remain free of a diagnosis of MDD. Univariate analyses demonstrated that those with MDD were more likely to be female, less likely to be physically active, more likely to have diabetes complications at baseline and have higher CSS. Mean scores for the WHO-5, PAID and PHQ-9 were poorer in those with incident MDD compared with those who had never had a diagnosis of MDD. Regression analyses demonstrated that higher PHQ-9, lower WHO-5 scores and greater CSS were significant predictors of incident MDD. Significant predictors of PHQ-9 were baseline PHQ-9 score, WHO-5, PAID and CSS.

**Conclusion:**

This study demonstrates the importance of psychosocial factors in addition to physiological variables in the development of depressive symptoms and incident MDD in people with type 2 diabetes. Stressful life events, depressive symptoms and diabetes-related distress all play a significant role which has implications for practice. A more holistic approach to care, which recognises the interplay of these psychosocial factors, may help to mitigate their impact on diabetes self-management as well as MDD, thus early screening and treatment for symptoms is recommended.

## Introduction

It is now well-known that the prevalence of both diabetes and mental health problems is increasing rapidly, with a greater risk for depression in people with diabetes compared to those without (Nouwen, 2010; 2015; Lloyd *et al*., 2012; Roy and Lloyd, 2012; Mommersteeg *et al*., 2013; Salinero-Fort *et al*., 2018). Comorbid diabetes and depression are linked to increased mortality as well as a greater risk for developing other conditions including heart and kidney disease (Reeves *et al*., 2015; Novak *et al*., 2016; Naicker *et al*., 2017; Nouwen *et al*., 2019). In addition, the combination of diabetes and depression has a far greater impact on well-being compared with depression and other comorbid diseases (Moussavi *et al*., 2007). There is evidence that depression can be treated successfully with both psychological and pharmacological interventions in people with diabetes (van der Feltz-Cornelis *et al*., 2010; Nicolau *et al*., 2013); however, more research is required to clarify which factors predict the onset of this condition. In addition, there are extra challenges given that emotional distress may be present in people with diabetes due to difficulties with the sometimes overwhelming requirements of self-management which can, in turn, impact on and be influenced by depression (van der Feltz-Cornelis *et al*., 2010; Baumeister *et al*., 2012; Petrak *et al*., 2015). Indeed, our own research has demonstrated that both elevated levels of diabetes-related emotional distress and a history of depression were independently associated with major depressive disorder (MDD) (Lloyd *et al*., 2015; Lloyd *et al*., 2018). A systematic review has suggested that diabetes distress can be reduced with tailored interventions (Schmidt *et al*., 2018), although it may be difficult to disentangle diabetes distress from depressive symptoms (Snoek *et al*., 2015). Longitudinal studies investigating risk factors for the incidence of clinically diagnosed depression in people with diabetes are few and far between; this paper reports the follow-up results from the INTERPRET-DD study which now has data collected in 12 countries (Lloyd *et al*., 2015); a study that is unique as it includes both a clinical diagnosis of depression and depressive symptoms as well as a measure of diabetes-related emotional distress, carried out in countries with vast differences in culture and health systems. We aimed to identify specific risk factors for the onset of diagnosed depression as well as depressive symptoms in this cohort of individuals with type 2 diabetes.

This study was initiated within the framework of the Dialogue on Diabetes and Depression (http://diabetesanddepression.org/), an international alliance of associations and organisations and continued under the auspices of the Association for the Improvement of Mental Health Programmes, a non-governmental organisation in Geneva engaged in educational and other programmes dealing with the comorbidity of mental and physical disorders (Sartorius and Cimino, 2012).

## Materials and methods

A full description of our protocol is provided in our earlier papers (Lloyd *et al*., 2015; Lloyd *et al*., 2018). In brief, between September 2013 and May 2015, a sample of consecutive out-patient clinic attenders with type 2 diabetes at each of the study sites was invited to participate in the study, with the aim of including 200 people with type 2 diabetes in each country. The teams of investigators in the collaborating sites (recruited from leading centres of excellence in their country) included at least one psychiatrist and one endocrinologist in each country. The treating physician or diabetologist in the diabetes clinic invited individuals to participate in the study. Diabetes clinics were based in either secondary or tertiary care centres, depending on the facilities available in each country. Written informed consent was obtained for participation in both the baseline and follow-up data collections.

### Inclusion/exclusion criteria

Eligible study participants were adults (18–65 years of age) with type 2 diabetes diagnosed at least 12 months before the point of contact attending their diabetes out-patient facilities. Individuals were excluded if they had been diagnosed with type 2 diabetes for less than 12 months as it is usual to experience a period of adjustment when first diagnosed. Other exclusion criteria were a diagnosis of type 1 diabetes, being unable to complete the survey tools due to communication or cognitive difficulties or having any life-threatening or serious conditions (e.g. cancer, stroke in the last 6 months). Those currently admitted or planning an admission for in-patient care to a hospital (unless admitted for diabetes self-management) were excluded as this group may have been receiving more intensive or different treatment for their diabetes and so were less comparable to those not admitted. Women who were pregnant/had had a child in the last 6 months were also excluded, as were those who had received a clinical diagnosis of dependency on alcohol or other substance (not tobacco) or a diagnosis of schizophrenia.

Prior to interview, the site investigators completed an information form for each eligible individual. This form included information from medical records, such as age, duration of diabetes, family history of diabetes and presence/history of diabetes complications. The latter included cardiovascular disease, retinopathy, peripheral neuropathy, peripheral vascular disease and renal disease and associated disorders. Most recent measurements of blood pressure, height and weight were also recorded. A diagnosis of Major Depressive Disorder (MDD) was made at interview so that the proximity of clinical records did not differ. Participants were asked if they lived in what they considered to be a rural or an urban area and reported their highest level of education (defined as no formal, some/completed primary, some/completed secondary school, or higher education which was defined as any college, post-graduate or professional training). Marital status was defined as married/cohabiting *v.* being single/widowed/divorced which, for our analyses, was dichotomised into living alone *v.* not living alone. Participants were also asked if they considered that they had a regular income.

Each participant was asked to complete the Patient Health Questionnaire (PHQ-9), the WHO-5 Well-being questionnaire and the Problem Areas in Diabetes Scale (PAID). The PHQ-9 consists of nine items on a four-point Likert-type scale (Kroenke and Spitzer, 2001). It has good sensitivity and specificity with regard to identifying cases of depression as well as being sensitive to change over time, and it has been used in a number of different countries (Kroenke and Spitzer, 2001). The WHO-5 is a five-item scale measuring feelings of well-being (World Health Organization, regional office for Europe, 1998). Unlike other scales, it identifies on a six-point scale the absence of positive feelings rather than the presence of negative ones (World Health Organization, regional office for Europe, 1998). The PAID is a 20-item questionnaire which measures the extent of diabetes-related emotional distress (Polonsky *et al*., 1995). Items include ‘feeling overwhelmed with your diabetes’ and ‘feelings of guilt or anxiety when you get off track with your diabetes management’. Moderate–severe levels of diabetes-related distress are defined as scores (standardised to 100) >40 (Polonsky *et al*., 1995). All questionnaires were completed using standard self-complete methods in the appropriate language, or assisted one-to-one collection, with the questions read out by the researcher and answered by the participant. Where no existing translation/cultural adaption of the questionnaires were available, they were adapted using standard forward/back translation procedures. In addition, each country's investigators ensured they were culturally applicable through their development over several iterative stages involving discussion and testing with a range of health care professionals and people with type 2 diabetes, focusing on the meaning of terms as well as language.

In order to diagnose MDD, a psychiatric interview was subsequently conducted by a trained interviewer using the MINI International Neuropsychiatric Interview (MINI; V5 or V6 depending on current psychiatric practice at the study site) (Sheehan *et al*., 1998). The MINI has been widely used in a range of different populations – including those with serious illnesses and in community surveys and is a reliable tool indicating the diagnostic category according to DSM-5 criteria (American Psychiatric Association, 2013). Individuals diagnosed with MDD (or other psychiatric disorders such as anxiety disorders) were advised to consult their physician for further assessment and treatment. If any individual indicated suicidality (question 9 on the PHQ-9), the psychiatrist conducting the clinical interview initiated immediate appropriate care.

After a minimum of 12 months, baseline respondents were contacted to participate a second time. The same instruments were used with the addition of the Holmes and Rahe Perceived Social Readjustment (HRPSR) Scale (Holmes and Rahe, 1967). The HRPSR measures stress in terms of life events that are hypothesised to increase the risk of illness (Holmes and Rahe, 1967). Individual scores for life events are weighted according to the perceived level of stress each one might engender. An additional measure was added to account for the degree of upset experienced if an event had occurred, measured on a four-point scale from 0 (not at all) to 3 (very much). This scale was added because the original HRPSR scale assumes that the felt impact of an event is similar regardless of context; our addition of the upset scale gave us a deeper insight into the experience of an event within the individual's overall life context. A composite stress score (CSS) was created that combined the experienced stressful events during the follow-up period with how much they upset the person, calculated as the product of occurrence and affect scores. Two additional items, ‘civil unrest’ and ‘physical, sexual, or emotional abuse’, were added to the life event list. A final unspecified item was added to allow for major life events that were not on the list to be recorded.

Change in MDD status between baseline and follow-up data collection was defined as (1) no MDD at either baseline or follow-up (or during the 3 years prior to baseline, or any time between baseline and follow-up), (2) MDD diagnosed at both baseline (or during the 3 previous years) and follow-up, (3) incident MDD, i.e. the participant reported at least one episode of depression between baseline and follow-up and no depression at baseline or during the previous 3 years, and (4) remission of depression, i.e. MDD diagnosed at baseline or up to 3 years prior and no depression between baseline and follow-up interview. The data reported here pertain only to those who fully participated and who therefore had complete information on socio-demographics, clinical and psychological status at both time points (*n* = 1616).

### Ethical approval

All procedures were approved by the ethics committees in each study setting, complied with the ethical standards of the relevant national and institutional committees on human experimentation and with the Helsinki Declaration of 1975 as revised in 2008, and included participation at both baseline and follow-up data collection. In addition, ethical approval was obtained from the Human Research Ethics Committee at The Open University, UK, where the data were stored for analysis.

### Statistical analysis

SPSS version 23 (Hosmer and Lemeshow, 2000) was used to analyse the data. For this analysis, we only selected those participants for whom we had data at both baseline and follow-up points and compared those with incident MDD to those with no MDD at either baseline or follow-up.

In order to examine the predictors of incident MDD during the follow-up period, we first carried out univariate analyses on the following baseline variables: country, marital status (living with someone *v.* living alone), level of education, income, location (urban *v.* rural area), insulin status, smoking status, exercise, sex, baseline complications, BMI, diabetes duration, age, WHO-5, PAID, PHQ-9, CSS as well as follow-up duration. The statistically significant (*p* < 0.05) variables were then entered into a bivariate logistic regression. The outcome variable was onset of MDD (no MDD at baseline and a diagnosis of MDD at follow-up) with the predictor variables being insulin, exercise, sex, baseline complications, age, WHO-5, PAID, PHQ-9 and the CSS. In step 1, we adjusted for country, and in step 2, we added the remaining variables with a forward step selection method.

In order to investigate whether self-report of depressive symptoms would yield similar predictors as the diagnosis of depression, a linear regression was carried out, using follow-up PHQ-9 score as the outcome variable and controlling for country in step 1, followed by a forward selection method for the predictor variables above in step 2.

## Results

A total of 1616 participants had known MDD status at both baseline and follow-up. Participation rates differed significantly by country ranging from 55.6% in India to 98.5% in Ukraine, and level of education (no formal education *v.* primary, secondary, higher education; 6.4 *v.* 10.0, 19.2 *v.* 22.0, 33.4 *v.* 10.4 and 29.5 *v.* 27.7%; *p* < 0.01). Individuals on insulin were more likely to participate in the follow-up data collection compared with those not on insulin (77.6 *v.* 72.0%; *p* < 0.01). Smokers were more likely to participate than non-smokers (81.0 *v.* 73.2%; *p* < 0.01). Diabetes distress scores were higher in participants compared to non-participants (17.9 ± 18.4 *v.* 16.1 ± 18.3; Mann–Whitney test *p* < 0.05), and the number of complications was higher in participants than non-participants (1.05 ± 1.38 *v.* 0.81 ± 1.04; *p* < 0.001); however, PHQ-9 scores did not differ significantly between those who participated in the follow-up and those who did not.

Table 1 shows the MDD status of all those with a psychiatric assessment at both baseline and follow-up with 120 (7.4%) incident MDD cases and 1317 (81.5%) continuing to remain free of a diagnosis of MDD. A surprisingly high rate of incident MDD was apparent in the Ukraine sample, whereas there were no new cases of MDD in the Ugandan sample. Very low incident rates were observed in the Russian, Polish and Kenyan participants. Univariate analyses comparing those who did not develop MDD with those who did (Table 2) found that the mean length of follow-up was the same; however, they were more likely to be female and taking insulin for their diabetes (Table 2). They were also less likely to have reported regularly taking part in physical activity and were more likely to have diabetes complications at baseline. Mean age in this latter group was somewhat lower than those who did not develop depression. Mean scores for the WHO-5, PAID and PHQ-9 were poorer in those with incident MDD or who had received a diagnosis of MDD at both time points compared with those who had never had a diagnosis of MDD. In addition, CSS were significantly higher in those with incident MDD compared to the other group (Table 2).

**Table 1.** Country data for those with depression status known at both baseline and follow-up

Country (*N*)	Males *N* (%)	Age Mean (s.d.)	Diabetes duration Mean (s.d.)	No MDD diagnosis within the time points (No–No) *N* (%)	Incident depression (No–Yes) *N* (%)
Argentina (77)	41 (53.2)	57.1 (8.02)	10.6 (6.9)	64 (83.1)	5 (6.5)
Germany (83)	51 (61.4)	54.4 (7.3)	7.0 (7.8)	72 (86.7)	6 (7.2)
India (105)	57 (54.3)	52.8 (9.7)	8.2 (5.8)	92 (87.6)	10 (9.5)
Italy (161)	94 (58.4)	56.8 (6.8)	10.0 (7.8)	138 (85.7)	8 (5.0)
Kenya (148)	32 (21.6)	50.0 (11.7)	7.2 (6.0)	131 (88.5)	5 (3.4)
Mexico (165)	63 (38.2)	52.4 (8.5)	11.1 (7.5)	127 (77.0)	9 (5.5)
Pakistan (148)	66 (44.6)	51.4 (9.0)	8.4 (6.0)	122 (82.4)	16 (10.8)
Poland (143)	74 (51.7)	57.6 (7.5)	10.0 (7.4)	109 (76.2)	5 (3.5)
Russia (151)	33 (21.9)	56.5 (7.7)	8.7 (6.2)	116 (76.8)	3 (2.0)
Serbia (187)	82 (43.9)	58.5 (5.7)	9.3 (5.8)	155 (82.9)	7 (3.7)
Uganda (118)	40 (33.9)	50.7 (8.2)	6.7 (5.3)	118 (100.0)	0 (0.0)
Ukraine (130)	58 (44.6)	46.6 (11.9)	8.9 (6.6)	73 (56.2)	46 (35.4)
Total (1616)	691 (42.8)	53.8 (9.3)	8.9 (6.7)	1317 (81.5)	120 (7.4)

**Table 2.** Univariate analyses of predictors of incident MDD

Nominal variables, *χ*^2^, one-sided* test for significance			
	Remained not depressed (*N* = 1317) *N* (%)	Incident MDD (*N* = 120) *N* (%)	*p*-value
Male	595 (45.1)	42 (35.3)	0.024
On insulin	511 (38.7)	68 (57.1)	<0.001
Regular exercise	773 (59.1)	49 (41.2)	<0.001
Married/cohabiting	938 (71.1)	78 (65.5)	0.121
Regular income	1108 (84.0)	93 (78.2)	0.068
Formal education			
None	84 (6.4)	13 (10.9)	
Primary school	252 (19.1)	27 (22.7)	
Secondary school	590 (44.7)	43 (36.1)	
Higher education	393 (29.8)	36 (30.3)	0.058
Urban location	1108 (84.0)	106 (89.1)	0.088
Smokers	196 (14.9)	19 (16.0)	0.415
Linear variables, ANOVA, one-sided test for significance			
	Remained not depressed Mean (s.d.)	Incident MDD Mean (s.d.)	*p*-value
Age	53.8 (9.5)	51.6 (9.6)	0.015
Diabetes duration	8.7 (6.7)	9.9 (7.5)	0.122
WHO-5 score	17.7 (4.9)	12.6 (5.4)	<0.001
Total PAID score	14.7 (16.0)	29.5 (18.4)	<0.001
PHQ-9 score	3.6 (3.5)	9.1 (4.7)	<0.001
Composite stress score	10.0 (13.4)	29.8 (21.6)	<0.001
Number of complications	0.9 (1.3)	1.9 (1.8)	<0.001
Body mass index	29.5 (6.1)	29.8 (5.7)	0.623
Follow-up time (months)	14.0 (2.4)	13.7 (1.9)	0.130

* One-sided significance levels were used because previous research has shown these factors to be implicated in the development and maintenance of depression.

A binary logistic regression analysis using remained non-depressed (i.e. no diagnosis at either time points) *v.* incident MDD as the binary outcome variable demonstrated that, after adjusting for country of study, higher PHQ-9 and lower WHO-5 scores at baseline and higher CSS during the follow-up period were all significant predictors of incident MDD (see Table 3).

**Table 3.** Significant predictors of incident depression diagnosis (MDD) in bivariate logistic regression, adjusted for country

Predictor	OR	95% CI	*p*-value
Baseline PHQ-9 score	1.23	1.15–1.31	<0.001
WHO-5 score	0.92	0.87–0.98	0.01
Composite stress score	1.06	1.04–1.09	<0.001

Outcome variable: depression (MDD) status at follow-up (no depression *v.* incident MDD).

*N* = 1364. Nagelkerke's *R*^2^ = 0.438. Non-significant factors: diabetes complications, PAID, sex, insulin, age, exercise.

A similar analysis using follow-up PHQ-9 score as the outcome variable found that, after adjusting for country (and excluding baseline PHQ-9 score from the analysis), WHO-5, PAID, CSS, sex and insulin treatment were significant predictors (Table 4). When baseline PHQ-9 score was added to the model (Table 5), the effect of sex and insulin was no longer significant. Therefore, in addition to explaining more of the variance (Adjusted *R*^2^ = 0.495 *v.* 0.419), the latter model was more parsimonious. Since heteroscedasticity was confirmed in the raw data, which tends for the model to underestimate *p*-values, a log-transformation was carried out. Results confirmed the significance of predictors above, with sex re-entering the model.

**Table 4.** Significant predictors of follow-up PHQ-9 scores in linear regression, adjusted for country (baseline PHQ-9 scores not entered as predictor variables)

Predictor	Standardised coefficient	*p*-value
WHO-5 score	−0.29	<0.001
PAID score	0.22	<0.001
Composite stress score	0.30	<0.001
Sex	0.05	0.011
Insulin	0.04	0.047

*N* = 1542. Adjusted *R*^2^ = 0.419. Non-significant factors: diabetes complications, PAID, age, physical activity.

**Table 5.** Significant predictors of follow-up PHQ-9 scores in linear regression, adjusted for country and including baseline PHQ-9 scores

Predictor	Standardised coefficient	*p*-value
Baseline PHQ-9 score	0.43	<0.001
Composite stress score	0.25	<0.001
PAID score	0.10	<0.001
WHO-5 score	−0.06	0.038

*N* = 1531. Adjusted *R*^2^ = 0.495. Non-significant factors: sex, insulin, age, complications, physical activity.

**Fig. 1.**
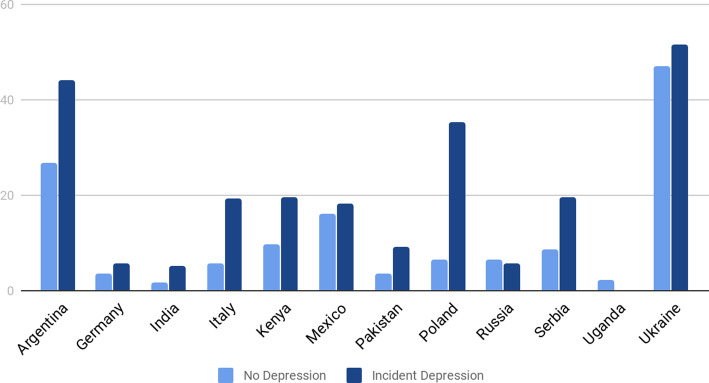
Composite stress scores by depression status for each country (for participants with a completed Holmes and Rahe).

PAID scores at both baseline and follow-up were significantly correlated with CSS (*R*^2^ = 0.374 and 0.512; *p* = 0.01, respectively). PAID and CSS did not significantly differ according to sex. Figure 1 shows the mean CSS for those who remained non-depressed and those with incident MDD by country.

Overall, the highest CSS were ‘death of a close family member’, ‘change in financial state’, ‘major change in the health of a family member’, ‘major personal injury or illness’, ‘change in sleeping habits’ and ‘change in eating habits’. Mexico reported the highest CSS for ‘major personal injury or death’, with Ukraine participants reporting the highest scores for ‘change in sleeping habits’ and ‘major change in the health of a family member’. Table 6 provides an overview of the most important stressors by country.

**Table 6.** Country stressors in order of importance (first six)

Argentina	Death of close family member (141)	Major personal injury or illness (113)	Major change in health of family member (99)	Change in sleeping habits (88)	Change in eating habits (87)	Death of a close friend (78)
Germany	Major personal injury or illness (60)	Death of close family member (46)	Change in financial state (21)	Major change in health of family member (17)	Vacation (17)	Change in responsibilities at work (15)
India	Death of close family member (46)	Marriage (16)	Other life event/stressor not specified elsewhere (14)	Religious or other cultural festival/activity approaching (11)	Change in financial state (10)	Gain of a new family member (10)
Italy	Death of close family member (96)	Major change in health of family member (90)	Death of a close friend (79)	Change in financial state (74)	Major personal injury or illness (73)	Vacation (66)
Kenya	Change in financial state (204)	Death of close family member (152)	Death of a close friend (144)	Change in sleeping habits (120)	Major change in health of family member (101)	Change in social activities (94)
Mexico	Death of close family member (326)	Major personal injury or illness (301)	Change in eating habits (267)	Trouble with in laws or other family member (136)	Major change in health of family member (133)	Gain of a new family member (126)
Pakistan	Change in social activities (60)	Change in number of family get-togethers (46)	Sexual difficulties (44)	Change in number of arguments with spouse (40)	Change in sleeping habits (40)	Death of close family member (35)
Poland	Major change in health of family member (110)	Change in sleeping habits (87)	Death of close family member (82)	Change in financial state (71)	Change in recreations (57)	Other life event/stressor not specified elsewhere (53)
Russia	Major change in health of family member (162)	Major personal injury or illness (127)	Change in financial state (90)	Change in sleeping habits (77)	Death of close family member (56)	Change in recreations (56)
Serbia	Change in financial state (212)	Religious or other cultural festival/activity approaching (194)	Vacation (133)	Major personal injury or illness (127)	Major change in health of family member (95)	Change in recreations (91)
Uganda	Major personal injury or illness (68)	Sexual difficulties (30)	Change in sleeping habits (29)	Change in financial state (20)	Other life event/stressor not specified elsewhere (14)	Change in eating habits (12)
Ukraine	Physical, sexual or emotional abuse (379)	Change in financial state (369)	Change in sleeping habits (367)	Sexual difficulties (313)	Major change in health of family member (310)	Change in eating habits (301)

The events rated as most upsetting were ‘death of a spouse’, ‘physical, sexual, or emotional abuse’, ‘divorce’, ‘marital separation’, ‘other life event/stressor not specified elsewhere’, ‘fired at work’ and ‘detention in jail or other institution’. The events rated the least upsetting were ‘marriage’, ‘religious holiday’, ‘vacation’, ‘outstanding personal achievement’, ‘pregnancy’ and ‘gain of a new family member’.

## Discussion

This study has demonstrated the importance of psychosocial factors as risk factors for the onset of MDD and increased depressive symptoms. The World Health Organisation has identified the treatment of depression and other mental health problems as a priority for the 21st century; however, only a few studies have identified the risk factors that are associated with the development of mental health problems, in particular in people with co-occurring conditions such as diabetes. Exceptions to this include studies in the Netherlands and the USA (Naranjo *et al*., 2011; Nefs *et al*., 2012; Johnson *et al*., 2014), with Nefs *et al.* identifying stressful life events (although only measured by a single item), diabetes complications and a previous history of depression as risk factors for subsequent depression (Nefs *et al*., 2012). Although our incident rate for MDD was slightly lower than the Netherlands study, this may be due to a different measurement tool and lack of a clinical interview to diagnose depression in that study. We have observed differences in the incidence of MDD according to country and there are a number of factors that may explain this. First, there are differences in psychosocial conditions, the clinical manifestation of depression and the nature of care provided in different countries. Second, the different gender and age composition of patients by country (particularly in Russia) could play a meaningful role.

### The role of stress

Although it is known that there is a link between diabetes and depression, the role of stress is less clear possibly due to differences in its measurement (Joseph and Golden, 2017). A few studies have reported a link between stress and diabetes self-management (Smith *et al*., 2020), but the impact of stressors not only in relation to diabetes but as part of the individual's broader experience has been less examined. A caveat to this has been the work proposing the concept of ‘syndemics’ which points to a complexity of factors that increase the risk of poor health (Mendenhall *et al*., 2015). Mendenhall *et al.* have observed that the prevalence of diabetes varies in different socio-economic groups, with co-existing stress and struggles living with diabetes greater in the poorest individuals. Acknowledging the cumulative effect of life stress and managing diabetes on mental health can provide a more holistic picture of the individual which has important implications for practice (Fisher *et al*., 2001).

Our adaptation of the Holmes and Rahe scale to include a measure of ‘upset’ has meant that we are able to measure stress in a way that is more likely to represent the actual perceived impact of events within the context of peoples' lives. Our measure does not make any assumptions as to the severity of an event, but rather it is based on the individual's perception once it occurs. In addition, we added specific events that were more culturally relevant to each country (as identified by the country-specific investigative team), to make our data more culturally relevant and country-specific. At the same time, we can identify the most common stressors, some of which challenge our ideas as to what are the most stressful events, such as a change in eating or sleeping habits. These latter events could be related to the physiological aspects of diabetes as well as to depression, especially given the strong correlation between stress and diabetes distress in our study. Our adapted scale could be a useful instrument to screen for vulnerability to depression in future studies.

### Cultural factors and risk of depression among people with type 2 diabetes

Culturally specific concerns with regard to variations in reporting depressive symptoms have been suggested (Kirmayer *et al*., 2017) and culturally adapted measurement instruments recommended (Tarricone *et al*., 2012). We have been able to mitigate this by ensuring that we utilised country-specific translations/interpretations for both the PHQ-9 and the MINI and that trained psychiatrists were from the same country/region and were able to ensure that the questions were asked in an appropriate way. A further strength of our study is the use of both a symptom scale and a diagnostic interview to determine the presence/absence of depressive symptoms/MDD.

Our finding that baseline PHQ-9 scores were significantly associated with incident MDD as well as depressive symptoms supports previous work on the association between these two measures, although unlike our prospective study in 12 countries, most research has been cross-sectional in single countries (Darwish *et al*., 2018). Recognition of depressive symptoms as a precursor to clinical levels of depression could lead to earlier interventions that reduce the risk of MDD. Although PAID (diabetes distress) scores were not found to be an independent predictor of MDD, our multivariable analysis found it to be a further important predictor of depressive symptomatology (PHQ-9). This is unsurprising, given their known association; however, it provides another potential avenue for the identification of those at risk of depression easily determined during clinical encounters (Schmitz *et al*., 2014; Darwish *et al*., 2018).

A limitation of our study is that only those who had complete data could be included in our analyses and were more likely to have complications, treated with insulin, smoke and have higher diabetes distress scores (although baseline PHQ-9 scores did not differ significantly). These individuals may have been more likely to return to their diabetes centre for care and so were more likely to have been available for follow-up recruitment.

Our findings have implications for clinical practice and the possibilities for reducing the risk of depression. The high (and increasing) prevalence of type 2 diabetes may induce healthcare providers to standardise the clinical management of this condition, in order to reduce professionals' time expenditure and burden to a minimum. Our data show that a more holistic approach, including a discussion of life events, may help to identify individuals at risk of developing psychological distress. Although it may initially increase consultation time, encouraging people with diabetes to discuss stressful events and providing emotional support may help to mitigate the risk of depression, the diagnosis of which has economic implications both at the individual and wider (health system) level. Given that stress and diabetes-related distress are correlated, by mitigating stress, diabetes distress may also be reduced, leading to a positive impact on the well-being of people with diabetes. Given that PHQ-9 scores are a predictor of MDD, early screening could prevent more severe outcomes.

## Supplementary material

For supplementary material accompanying this paper visit http://dx.doi.org/10.1017/S2045796020000438. click here to view supplementary material

## Data

The data will not be available at the present time due to it being analysed for country-specific publication in accordance with the INTERPRET-DD investigators' agreement.
