# Supplementary material for: Factors associated with the onset of major depressive disorder in adults with type 2 diabetes living in 12 different countries: results from the INTERPRET-DD prospective study
Source: Epidemiol Psychiatr Sci. 2020 Jun 2;29:e134. doi: 10.1017/S2045796020000438 (PMC7303789; doi:10.1017/S2045796020000438)
Supplement: Supplementary file 1 [file S2045796020000438sup001.doc]

**Supplementary Table 1**

**Psychometric Scales - follow-up by Country**

**Supplementary Table 2**

**Psychometric Scales - follow-up by Sex**

**Supplementary Table 3**

**Psychometric Scales - follow-up by Country and Sex**
